# Evaluating Single Spacecraft Observations of Planetary Magnetotails With Simple Monte Carlo Simulations: 2. Magnetic Flux Rope Signature Selection Effects

**DOI:** 10.1029/2018JA025959

**Published:** 2018-12-22

**Authors:** A. W. Smith, C. M. Jackman, C. M. Frohmaier, R. C. Fear, J. A. Slavin, J. C. Coxon

**Affiliations:** ^1^ Department of Physics and Astronomy University of Southampton Southampton UK; ^2^ Institute of Cosmology and Gravitation University of Portsmouth Portsmouth UK; ^3^ Climate and Space Sciences and Engineering University of Michigan Ann Arbor MI USA

**Keywords:** flux ropes, Mercury, MESSENGER, Monte Carlo, reconnection, magnetotail

## Abstract

A Monte Carlo method of investigating the effects of placing selection criteria on the magnetic signature of in situ encounters with flux ropes is presented. The technique is applied to two recent flux rope surveys of MESSENGER data within the Hermean magnetotail. It is found that the different criteria placed upon the signatures will preferentially identify slightly different subsets of the underlying population. Quantifying the selection biases first allows the distributions of flux rope parameters to be corrected, allowing a more accurate estimation of the intrinsic distributions. This is shown with regard to the distribution of flux rope radii observed. When accounting for the selection criteria, the mean radius of Hermean magnetotail quasi‐force‐free flux ropes is found to be $589_{-269}^{+273}$ km. Second, it is possible to weight the known identifications in order to determine a rate of recurrence that accounts for the presence of the structures that will not be identified. In the case of the Hermean magnetotail, the average rate of quasi‐force‐free flux ropes is found to 0.12 min^−1^ when selection effects are accounted for (up from 0.05 min^−1^ previously inferred from observations).

## Key Points

- A Monte Carlo method is presented to estimate and correct for sampling biases in spacecraft surveys of magnetic flux ropes
- Method allows the correction of observed distributions (e.g., flux rope radii) according to the selection criteria employed
- Accounting for unidentified flux ropes increases the average rate of flux ropes in Mercury's magnetotail from 0.05 to 0.12 min^−1^

## 1 Introduction

Magnetic reconnection is the process by which two adjacent magnetic regimes can interact and reconfigure. The process itself occurs on the scale of the gyroradius of ions and electrons, however it can result in the formation of much larger magnetic structures, such as magnetic flux ropes. The radius of these structures can range from several times the ion scale (e.g., Eastwood et al., 2016; Teh et al., 2017) up to hundreds or thousands of kilometers (e.g., Ieda et al., 1998; Moldwin & Hughes, 1992; Sibeck et al., 1984; Slavin et al., 1989, 1995), significant fractions of planetary radii. Flux ropes are thought to form between adjacent sites of reconnection (often termed x‐lines) in the magnetotail current sheet (e.g., Slavin et al., 2003). Once formed, the magnetic flux ropes will be ejected along the current sheet away from the dominant reconnection site (often termed the neutral line). Flux ropes are also observed on the dayside magnetopause, and termed flux transfer events (FTEs). Suggested FTE formation mechanisms include patchy (Russell & Elphic, 1978) or bursty reconnection (Scholer, 1988), in addition to the multiple x‐line model (Lee & Fu, 1985).

If a flux rope happens to pass over a spacecraft, a distinctive magnetic signature will be recorded: a smooth rotation of the magnetic field accompanied by a strong enhancement of the field in the axial direction of the flux rope (e.g., Hughes & Sibeck, 1987; Moldwin & Hughes, 1991). The orientation of the field deflection allows the direction of travel of the flux rope to be inferred, and therefore the location of the neutral line relative to the spacecraft. In addition, the location and recurrence of magnetic flux ropes can allow the determination of the type of conditions that are favorable for reconnection onset.

After their creation, adjacent flux ropes may merge through what has been termed “secondary reconnection,” evidence for which has been observed in the magnetotail (Retinò et al., 2008; Wang et al., 2016; Zhao et al., 2016) and on the magnetopause (Zhou et al., 2017). One of the predictions of the coalescence model of flux rope growth is that the distribution of flux rope sizes (at larger radii) can be approximated by a decaying exponential. It has been found that the size distribution of FTEs is a good fit to this model at large radii (*r*>∼4,000 km); while inconsistent decreases in the distributions at lower radii have been attributed to instrumental and identification limitations (Fermo et al., 2010, 2011). A recent study of subsolar FTEs highlighted the importance of correcting for the relative impact parameter of the spacecraft; without this correction the distribution returns an underestimate of the mean radius (Akhavan‐Tafti et al., 2018).

The location and properties of magnetotail flux ropes are often investigated through large statistical surveys of in situ spacecraft data at Earth (e.g., Borg et al., 2012; Imber et al., 2011; Moldwin & Hughes, 1992; Slavin et al., 2003), Mercury (e.g., DiBraccio et al., 2015; Smith, Slavin, Jackman, Poh, et al., 2017; Sun et al., 2016), and Mars (e.g., Briggs et al., 2011; Vignes et al., 2004). However, these surveys of in situ data are fundamentally limited by both the orbital locations of the spacecraft and also any criteria placed upon the signatures of the flux ropes required. We investigate the effect of orbital sampling in a companion paper (Smith et al., 2018); while in this study we investigate the effect of placing selection criteria on the magnetic field signature.

Criteria are often placed on the magnetic field signatures of flux ropes in order to distinguish events from other magnetospheric phenomena, for example, Alfvénic waves (e.g., Slavin et al., 1989). However, placing specific limitations on the signature required will preferentially select a subset of the underlying population, the impact of which can be difficult to quantify. A commonly used criterion in magnetotail surveys is a fixed lower limit on the magnitude of the field deflection required (i.e., in the north‐south field component; e.g., Ieda et al., 1998; Moldwin & Hughes, 1992; Sun et al., 2016). More recently, in an attempt to identify smaller scale events, criteria have been developed that require deviation above the level of background fluctuations of the field, particularly at Jupiter (Vogt et al., 2010), Saturn (Jackman et al., 2014; Smith et al., 2016), and Mercury (Smith, Slavin, Jackman, Fear, et al., 2017). Criteria can also be placed upon the enhancements observed in the axial direction and the total field (e.g., Sun et al., 2016). Meanwhile, some time‐based criteria are explicitly selected or enforced by the resolution of the data employed; identification schemes often require several data points and thus the cadence of the data will impose a lower limit to the duration of the signatures identified (e.g., Imber et al., 2011; Moldwin & Hughes, 1992; Smith, Slavin, Jackman, Poh, et al., 2017). In this work the criteria placed on the magnetic field will be discussed, however constraints may also be placed on the local plasma environment (e.g., density, temperature, or plasma beta) if such measurements are available (e.g., Ieda et al., 1998; Moldwin & Hughes, 1992).

### 1.1 Mercury's Magnetotail

During the M2 and M3 flybys of the MESSENGER (MErcury Surface, Space ENvironment, GEochemistry and Ranging) spacecraft several reconnection related structures were observed whose signatures lasted between ∼1 and 3 s (Slavin et al., 2012). In the absence of high cadence plasma data, an estimated ejection velocity of ∼500 km/s (the mean observed in the terrestrial magnetotail; Ieda et al., 1998; Slavin et al., 2003) translates these observations to diameters of between ∼500 and 1,500 km.

Later, MESSENGER orbited Mercury between March 2011 and April 2015 (Solomon et al., 2007), recording high resolution (20 vectors per second) magnetometer data (Anderson et al., 2007). DiBraccio et al. (2015) performed a survey of 122 plasma sheet crossings, identifying 49 flux ropes with an average duration of 0.74 s, shorter than that initially observed during the MESSENGER flybys. Assuming that the flux ropes traveled at approximately the average Alfvén velocity (465 km/s; DiBraccio et al., 2015), and correcting for the trajectory of the spacecraft, the average radius was found to be 345 km; much smaller than the previous observations. More recently, Smith, Slavin, Jackman, Poh, et al. (2017) identified 248 flux ropes using an automated procedure (Smith, Slavin, Jackman, Fear, et al., 2017), and recorded an average duration of 0.83 s. Approximately 30% (74) of the flux ropes were found to be well modeled by the cylindrically symmetric force‐free model. This allowed the approximate spacecraft trajectory through the structure to be modeled and the duration to be corrected for the relative impact parameter. Once more combining this with the average Alfvén velocity allowed the calculation of a mean radius: 262 km, again somewhat smaller than previous estimates. This reduction in mean radius was partially attributed to the detailed automated search method, and the subsequent selection of small scale, shorter magnetic signatures. Therefore, care must be taken to account for the identification procedure (and resulting sample of events) when discussing the statistical results of a survey.

Sun et al. (2016) performed a semiautomated survey of 98 plasma sheet crossings, using previous observations (DiBraccio et al., 2015; Slavin et al., 2012) to target flux rope magnetic field signatures with specially designed selection criteria. The 39 flux ropes were identified at an average rate of 0.022 min^−1^. Following this, Smith, Slavin, Jackman, Poh, et al. (2017) identified 248 flux ropes at a higher average rate of 0.05 min^−1^. Both surveys used different selection criteria, and thus an understanding of how these initial choices affect the inferred results is crucial.

In this work we investigate the effects that selection criteria will impose on statistical surveys: in the number and rate of structures observed and the inferred distributions of parameters (e.g., radius). The Monte Carlo‐based technique will be discussed in the following section, along with the magnetic field model utilized. This will be followed in section 3 by a discussion of the criterion employed by two recent surveys of MESSENGER spacecraft data in the Hermean magnetotail (Smith, Slavin, Jackman, Poh, et al., 2017; Sun et al., 2016). The method will then be used to estimate the underlying distributions from which the results of Smith, Slavin, Jackman, Poh, et al. (2017) were identified. Finally, the recurrence of flux ropes will also be discussed, using the technique to estimate the unseen or unidentified fractions of the population.

## 2 Model and Method

In this section the chosen flux rope model and the Monte Carlo method used to probe the effects of selection criteria are discussed.

### 2.1 The Force Free Model

A model is used to allow the transformation from the intrinsic properties of the flux ropes to the observable quantities (on which the constraints are generally placed). The observable quantities most commonly constrained include the duration of the magnetic signature, the magnitude of the field deflection (in the north‐south field component), and the size of the peak in the axial or total field. In this work we employ the cylindrically symmetric, constant *α* force‐free model (Burlaga, 1988; Lepping et al., 1990; Lundquist, 1950), corresponding to the lowest energy equilibrium state of helical magnetic fields (Priest, 1990). More complex models could be used in the future, and these may also allow additional parameters to be investigated.

**Figure 1:**
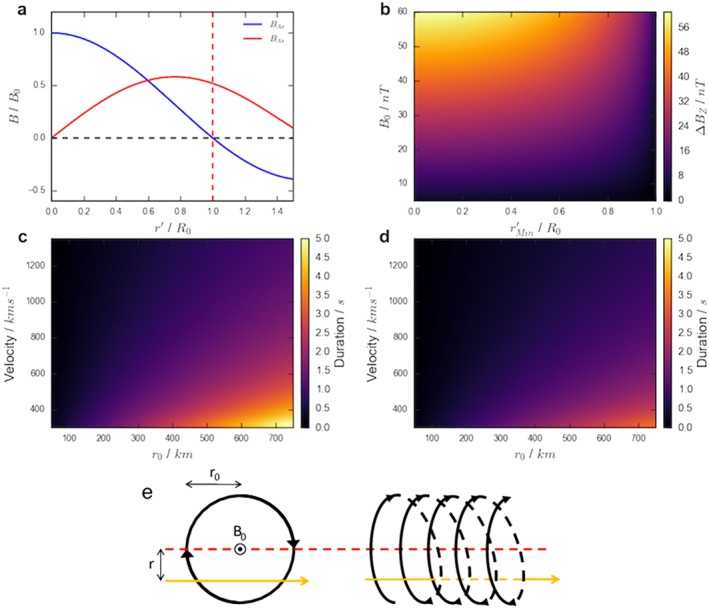
Figure illustrating the use of the force‐free model in transforming from intrinsic properties to observable quantities. Equations (1) and (2) are shown in panel (a). Panel (b) shows how the combination of core field and impact parameter combine to provide the field deflection in the B_Z_ component (given the assumptions in the text). Panels (c) and (d) show how the combination of flux rope velocity and radius combine to provide the duration of the magnetic signatures for $r^{\prime }$ = 0 and 0.75, respectively. Panel (e) shows two projections of an example spacecraft trajectory (yellow) though a model flux rope (where black lines indicate the magnetic field).

Principally, the model assumes that flux ropes can be assumed to be cylindrically symmetric and force‐free: that is, **J** × **B** = 0 and ∇*P* = 0 (where **J** is the current density, **B** is the magnetic field, and *P* is the thermal plasma pressure). Given these assumptions, the flux rope magnetic field in local cylindrical coordinates can be written as

(1) $$B_{\mathrm{Ax}}=B_{0}J_{0}(\alpha r^{\prime }),$$

(2) $$B_{\mathrm{Az}}=B_{0}HJ_{1}(\alpha r^{\prime }),$$

(3) $$B_{R}=0,$$

where *B*_0_ is the magnitude of the axial (or core) magnetic field, *J*_0_ and *J*_1_ are the zeroth‐ and first‐order Bessel functions, *H* is the helicity of the flux rope (*H* =± 1), and $r^{\prime }$ is the distance from the center of the flux rope in units of the flux rope radius ( $r^{\prime }=\frac{r}{r_{0}}$). If the constant (*α*) is set to 2.4048 then the configuration is such that the field is purely azimuthal at the edge ( $r^{\prime }=1$) and purely axial at the center ( $r^{\prime }=0$). This scenario is demonstrated (for equations (1) and (2)) in Figure 1a, where the field values are scaled to the strength of the core field (*B*_0_). Figure 1e shows two projections of a spacecraft trajectory through a model flux rope.

The model allows, with the application of some simple assumptions, that a combination of the following four intrinsic parameters will allow an estimation of the magnetic signature (e.g., duration and deflection size) of the flux rope encounter: velocity, radius (*r*_0_), core field (*B*_0_), and distance of closest approach to the flux rope axis (the impact parameter: $r_{\mathrm{Min}}^{\prime }$). First, if it is assumed that the flux rope is oriented such that the axial field is directed along the dawn‐dusk axis, then it follows that the field deflection in the north‐south field component will be solely due to the changing azimuthal component of the flux rope (**B**_*Az*_). From equation (2) it can be seen that the magnitude of the azimuthal component in the leading and trailing hemispheres of the flux rope ( $r^{\prime }=1$) will be solely determined by the value of the core field (|*B*_0_|). Meanwhile, the closest approach to the flux rope axis ( $r_{\mathrm{Min}}^{\prime }$), or the impact parameter, will control the orientation of the axial field relative to the north‐south unit vector. Therefore, combining the impact parameter ( $r_{\mathrm{Min}}^{\prime }$) and the core field strength will allow the calculation of the field deflection in the north‐south field component: Δ*B*_*Z*_. This is shown in Figure 1b. As may be expected, the largest field deflections are found for flux ropes with the strongest core fields (*B*_0_) that are encountered at small impact parameters ( $r_{\mathrm{Min}}^{\prime }$). It can be seen that the impact parameter has a relatively small effect up until around $r_{\mathrm{Min}}^{\prime }\geq 0.6\;r_{0}$, at which point the substantial change in the curvature of the field begins to considerably reduce the magnitude of the north‐south field deflection.

Second, if it is assumed that the relative velocity of the spacecraft is negligible and that the flux rope moves either planetward or tailward from its origin then a combination of its velocity, radius (*r*_0_), and the impact parameter ( $r_{\mathrm{Min}}^{\prime }$) will allow the determination of the duration of the magnetic signature of the flux rope (i.e., the duration of the peak to peak field deflection, sometimes known as the “characteristic time”; Kawano et al., 1992). This ignores any signature that may be created by the magnetic field draped around the flux rope. The duration of the magnetic signature is shown for combinations of flux rope radii and velocities for impact parameters of $r_{\mathrm{Min}}^{\prime }=0$ and $r_{\mathrm{Min}}^{\prime }=0.75$ in Figures 1c and 1d, respectively. It should be noted that Figures 1c and 1d are plotted with the same color scale for ease of comparison. As is intuitive, faster moving, smaller flux ropes produce shorter magnetic signatures and vice versa. Additionally, if the impact parameter is increased then the duration of the signature will be reduced (e.g., from comparing Figures 1c and 1d).

With this setup there is assumed to be no dawn‐dusk variation in the structure of the flux rope, such that it is approximated as a cylindrical object encountered at a tangent to its axial direction. Effects of limiting the extent of the structure in the azimuthal direction are considered in the companion study (Smith et al., 2018). Additionally, significant tilting of flux ropes away from the simple orientation considered above has been observed at the Earth (e.g., Kiehas et al., 2012; Slavin et al., 2003; Walsh et al., 2007) and Mercury (e.g., Smith, Slavin, Jackman, Poh, et al., 2017; Sun et al., 2016). Such tilting would have the effect of reducing the magnitude of the field deflection and the axial field in the *B*_*Y*_ component of the field: this in turn could impact the reported observation of flux ropes as their reduced field signatures may fall below detection thresholds. For this work we have started with the simplest set of assumptions which describe the system with a high degree of fidelity: in the future as observations advance and more is learned about the orientation of flux ropes, alterations and additions can be made to the model.

## 3 Evaluating Selection Bias

It is possible to simulate many thousands of flux rope encounters, randomly selecting combinations of flux rope radius (*r*_0_), core field strength (*B*_0_), velocity, and the impact parameter ( $r_{\mathrm{Min}}^{\prime }$) of the spacecraft trajectory. For each simulated flux rope encounter, the magnitude of the resulting field deflection (Δ*B*_*Z*_), the duration of the signature (Δ*T*), and the magnitude of the peaks in the axial and total field ( $B_{Y}^{\mathrm{Max}}$ and |*B*|^*Max*^) can be calculated (as demonstrated in Figure 1). It is then possible to compare these values to the selection criteria enforced by recent surveys. In some studies the values are required to be a certain level above background; when this is the case the background characteristics are randomly drawn from a set of 319 MESSENGER plasma sheet crossings (Poh et al., 2017).

For each combination of physical parameters it is possible to determine the fraction of flux ropes that would be identified by a given survey. This allows the recovery of combinations of parameters to be evaluated and any interdependence quantified. Below, two recent surveys of the Hermean magnetotail will be evaluated and compared (Smith, Slavin, Jackman, Poh, et al., 2017; Sun et al., 2016). Initially the four intrinsic parameters will be drawn from uniform distributions. One million random combinations were simulated.

### 3.1 Application to the Sun et al., 2016, Flux Rope Survey

Sun et al. (2016) performed a survey of 98 intervals during which MESSENGER crossed through the magnetotail plasma sheet. The MESSENGER magnetometer data were searched with an automated method, and the following criteria placed upon any field signature:

1. |Δ*B*_*Z*_|≥15*n* *T*,
2. $|B_{Y}^{\mathrm{Max}}|\geq \bar{\left|B_{Y}\right|}+5\;\mathrm{nT}$,
3. $|B{|}^{\mathrm{Max}}\geq \bar{\left|B\right|}+5\mathrm{nT}$,
4. 0.15*s*≤Δ*T*≤5*s*,

where $\bar{\left|B_{Y}\right|}$ and $\bar{\left|B\right|}$ are the average of the *B*_*Y*_ component and total field, respectively (for the time period from 0.5 s before the start of the event until 0.5 s after), while $\left|B_{Y}^{\mathrm{Max}}|\right|$ and |*B*|^*Max*^ are the absolute values of the peaks in the field. Checks were also performed upon the field rotation observed in minimum variance coordinates, and to ensure that the point of inflection (of the field rotation) was coincident with the peak in the axial field. These criteria have not been explicitly recreated in this study as they would not reject any of the signatures generated (due to the use of the force‐free model). In total, Sun et al. (2016) located 39 flux ropes.

**Figure 2:**
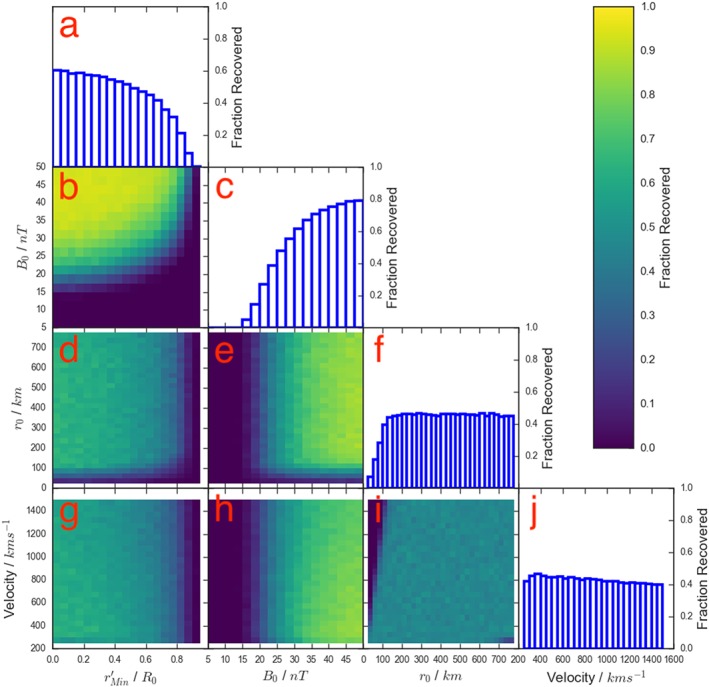
A four‐dimensional grid showing how the selection criteria of Sun et al. (2016) affects the fraction of flux ropes identified as a function of the intrinsic properties of the flux ropes. The diagonal panels show the fraction of flux ropes recovered as a function of the four intrinsic parameters (panels a, c, f, j). The six panels in the lower left show all combinations of the four parameters (panels b, d, e, g, h, i), while the color indicates the fractional recovery for flux ropes in that region of parameter space.

Figure 2 shows the effects of applying the Sun et al. (2016) criteria listed above to the randomly generated force‐free flux ropes (described above). The four intrinsic flux rope parameters are shown across the bottom, while a histogram of the fraction of flux ropes recovered for each is shown along the top of each column (diagonally: panels a, c, f, j). The six lower left panels then show the possible combinations of the parameters (panels b, d, e, g, h, i), with the color indicating the fraction of the generated flux ropes that were recovered given the selection criteria.

Figure 2a shows the recovery fraction as a function of the impact parameter. It can be seen that even if the spacecraft passes through the center of the flux rope ( $r_{\mathrm{Min}}^{\prime }=0$) only 60% of the generated flux ropes are identified. The recovery fraction can then be seen to drop off increasingly quickly as the impact parameter increases. There are two main contributing factors to this; the first is that structures with weak core fields will result in small deflections of the field (from quasi‐force‐free configurations). The second factor is that as the impact parameter increases (i.e., the spacecraft trajectory is further removed from the center of the structure), the magnitude of the north‐south field deflection will reduce due to the curvature of the flux rope: this will also have the effect of reducing the duration of the signature. The combination of these factors can be seen in Figure 2b, showing impact parameter against *B*_0_, where as the impact parameter increases a larger core field is required for identification. Figure 2d shows the reduction in recovery fraction for lower values of *r*_0_ (≤100 km) and higher values of $r_{\mathrm{Min}}^{\prime }$ (≥0.7*r*_0_), both of which result in a decreased duration of the field signature.

Figure 2c shows the recovery fraction as a function of core field, highlighting how the effectiveness of the survey decreases significantly below a core field of ∼20 nT. Therefore, if there is a significant fraction of the intrinsic population that possesses small core fields (≤20 nT), then they will be poorly represented by the identified sample. This has important consequences for the inferred rate of flux rope generation, and thereby magnetotail reconnection. Figures 2e and 2h are both dominated by the reduction in efficiency at low values of the core field.

The time criterion used by Sun et al. (2016) can be seen to relatively evenly sample the tested radius parameter space (Figure 2f), exhibiting at drop at only very small flux ropes (*r*_0_≤ 100 km). Meanwhile, Figure 2j shows that the velocity recovery is even for the range of values tested. The radius and velocity fractional recoveries are combined in Figure 2i, and appear fairly flat for the majority of the parameter space. The principle exception being small (*r*_0_≤ 100 km) flux ropes, for which there is a clear relation with the velocity (such that the product of the velocity and *r*_0_ must be greater than 0.15 s). If a statistical study is concerned with the relative shape of an observed distribution, then the shape of the recovery distribution is a fundamentally important property of the selection criteria adopted.

### 3.2 Application to the Smith et al., 2017, Flux Rope Survey

For comparison, Smith, Slavin, Jackman, Poh, et al. (2017) performed an automated search of 319 plasma sheet crossings (first identified by Poh et al., 2017). The following key selection criteria were placed upon the magnetic signatures:

1. Δ*B*_*Z*_≥1*σ* *B*_*Z*_,
2. $r_{\mathrm{Min}}^{\prime }\leq 0.5$,
3. 0.25*s*≤Δ*T*≤3*s*,

where *σ* *B*_*Z*_ is the local standard deviation of the *B*_*Z*_ component and $r_{\mathrm{Min}}^{\prime }$ is the impact parameter of the flux rope encounter. Several other criteria were also placed upon the signature; for full details the interested reader is directed to Smith, Slavin, Jackman, Fear, et al. (2017). The criteria based on the quality of model fit and results of the minimum variance analysis have not been included, they would not reject the model field signatures generated by the force‐free model. In addition, Smith, Slavin, Jackman, Fear, et al. (2017) employed a wavelet transform in order to locate peaks in the axial and total field; this has also not been recreated by the method. In total, Smith, Slavin, Jackman, Poh, et al. (2017) located 248 flux ropes, of which 74 were found to be well fitted by the force‐free model. The quasi‐force‐free subset will be used for comparison in section 4 (as their relative trajectory could be sufficiently well modeled).

**Figure 3:**
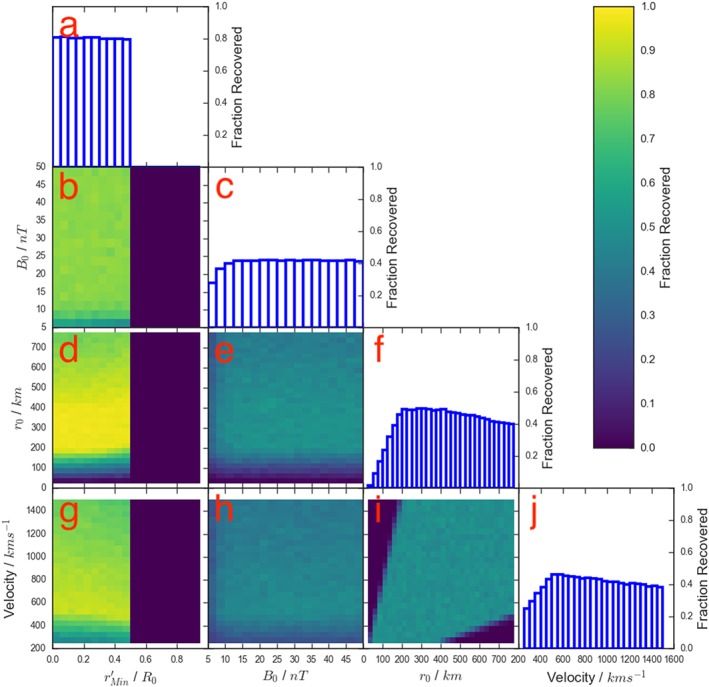
A four‐dimensional grid showing how the selection criteria of Smith, Slavin, Jackman, Poh, et al. (2017) affects the fraction of flux ropes recovered as a function of the intrinsic properties of the flux ropes. The format is as in Figure 2.

Figure 3 details the effects of the criteria above in the same format as Figure 2. One of the standout properties of Figure 3 is the impact of imposing the selection criterion regarding the impact parameter ( $r_{\mathrm{Min}}^{\prime }\geq 0.5$). This immediately reduces the fraction of flux ropes identified by a factor of two (as the impact parameter can be drawn from a uniform distribution). However, it can be seen that there is a fairly flat fractional recovery within this cut‐off in Figure 3a, in contrast to the shape of the fractional recovery shown in Figure 2a. This is a result of requiring the deflection to be greater than 1*σ* of the background; in practical terms this is approximately a factor of three reduction in threshold (1*σ*∼5 nT). The reduced threshold also leads to a larger peak fractional recovery as a function of impact parameter (∼0.8 compared to the ∼0.6 shown in Figure 2a). Figure 3c also shows a fairly flat recovery as a function of core field, once more in contrast to Figure 2c. Therefore, the criteria employed by Smith, Slavin, Jackman, Poh, et al. (2017) more evenly samples the population of flux ropes with small core fields (e.g., *B*_0_≤20*n* *T*), compared to the criteria used by Sun et al. (2016).

Figure 3i shows the recovery fraction projected onto the radius against velocity space and shows that both small, fast moving and large, slow moving flux ropes are poorly sampled by the Smith, Slavin, Jackman, Poh, et al. (2017) criteria. This has the result of distorting the recovery fraction distribution as a function of both radius and velocity (Figures 3f and 3j). This shape of fractional recovery distribution will have significant effects on the inferred distributions from the survey and should be taken into account when interpreting the results.

### 3.3 Interpretation and Limitations

There are several important factors to note when interpreting the results of Figures 2 and 3. The first is that the absolute magnitude of the recovery fractions is dependent upon the extent of the parameter space sampled (e.g., the lower limit of the core field, *B*_0_). If the parameter space were extended then it is likely that the additional flux ropes would be poorly recovered by the selection criteria (as they have not been designed to select those structures). This would have the effect of reducing all of the inferred fractional recoveries. Therefore, the absolute magnitudes of the recovery fractions should be interpreted with caution. To minimize the effects of overextending the parameter space, the limits were selected based upon the Hermean magnetotail flux ropes observed by previous works (e.g., DiBraccio et al., 2015; Smith, Slavin, Jackman, Poh, et al., 2017; Sun et al., 2016).

A second consideration is that the generation of the parameters is completely independent. However, if a pair of parameters were known to be correlated then this sampling may be unrepresentative. A related issue is the choice of distribution from which the parameters are drawn. In Figures 2 and 3, the four model parameters have been drawn from uniform distributions as a simple first approximation. If the absolute shape and parameters of the intrinsic distributions were known, then they should be used. However, as discussed above with regards to the extent of the parameter space, the main impact of the choice of distribution would be to change the absolute magnitudes of the recovery distributions. As an example, Fermo et al. (2011) suggested that the distribution of flux rope radii may follow an exponentially decreasing tail. This would imply that there are more small flux ropes than there are large, and so a uniformly distributed population will undergenerate small flux ropes. This has implications for the overall magnitudes of the recovery distribution: smaller flux ropes are less likely to be identified and so correcting for this effect would reduce the overall fraction recovered.

**Figure 4:**
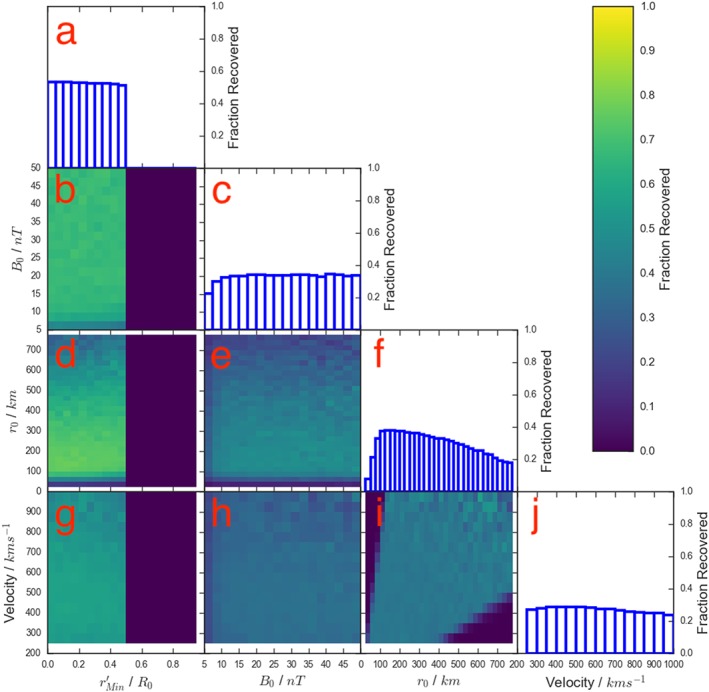
A four‐dimensional grid showing how the selection criteria of Smith, Slavin, Jackman, Poh, et al. (2017) affects the fraction of flux ropes recovered as a function of the intrinsic properties of the flux ropes. The format is as in Figures 2 and 3. However, the four parameters are now drawn from the following distributions: $r_{\mathrm{Min}}^{\prime }$ from a uniform distribution between 0 and 1; B_0_ from an exponential distribution with a mean of 22.5 nT; r_0_ from an exponential distribution with a mean of 250 km; and Velocity from a normal distribution with a mean of 450 ± 200 km/s. It should be noted that the upper end of the velocity range has been truncated (compared to Figure 3) due to poor sampling with the selected distributions.

To test this further, Figure 4 shows the selection criteria of Smith, Slavin, Jackman, Poh, et al. (2017) applied to flux ropes generated from distributions that are perhaps more representative of the intrinsic distributions. The impact parameter ( $r_{\mathrm{Min}}^{\prime }$) is still drawn from a uniform distribution; the relative distance of the spacecraft from the flux rope axis should be completely random. The flux rope radii are drawn from an exponential distribution with a mean of 250 km; the choice of distribution is consistent with modeling efforts (Fermo et al., 2010, 2011) and terrestrial magnetopause flux rope observations (Akhavan‐Tafti et al., 2018), while the mean is taken from a recent survey of Hermean magnetotail flux ropes (Smith, Slavin, Jackman, Poh, et al., 2017). The core field (*B*_0_) is also drawn from an exponential distribution with a mean of 22.5 nT; where the shape is consistent with previous Hermean magnetotail surveys (e.g., DiBraccio et al., 2015), and the mean is once more taken from a recent survey (Smith, Slavin, Jackman, Poh, et al., 2017). Finally, the velocity is drawn from a normal distribution with a mean of 450 km/s and a standard deviation of 200 km/s; this is consistent with terrestrial magnetotail studies (Moldwin & Hughes, 1992; Slavin et al., 2003) and also similar to the Alfvén velocity observed in the Hermean plasma sheet (DiBraccio et al., 2015).

Overall, comparing the results with the uniform source distributions (Figure 3) with those produced with the source distributions described above (Figure 4), the main result is that all of the recovery fractions have dropped by a factor of approximately two. This is a result of the increased sampling of those flux ropes with smaller radii (*r*_0_) and core fields (*B*_0_). As the source distributions used to produce Figure 4 are not fully constrained, for the remains of the study the recovery fractions with uniform distributions (i.e., Figure 3) will be used. For the majority of the later sections the shape of the distribution will be of more importance than the absolute magnitude of the recovery fraction. In section 4.2 the magnitudes are important, and therefore the estimates obtained for the intrinsic rate should be regarded as lower limits.

Finally, in all surveys, especially those undertaken automatically by algorithms, cuts must be made to distinguish the events of interest from other magnetotail phenomena. While this analysis can show the effects of the selection criteria on underlying populations (and potentially aid the placement of those limits), it only shows one factor that will affect the quality of the survey. For example, to maximize the derived recovery efficiencies it would be ideal to place no (or very low) thresholds. However, this would lead to a very large number of spurious or nuisance identifications which would inhibit or mislead the conclusions of the survey. Care must be taken therefore to balance these competing considerations, for example through the use of contingency tables and metrics such as the Heidke skill score (Heidke, 1926): a technique often used in space weather forecasting (e.g., Pulkkinen et al., 2013; Stephenson & Stephenson, 2000).

## 4 Applications

The evaluation of the fractional recovery of flux ropes (as a function of underlying parameters) enables further interpretation of survey results. This is particularly crucial where the surveys are compared with statistical results from modeling, investigations that are not subject to the same instrumental constraints. Below, the impact of selection effects will be evaluated when interpreting histograms of an observed property. The effects on the inferred rate of flux rope observations will then be investigated. The Smith, Slavin, Jackman, Poh, et al. (2017) catalog represents a much larger sample, complete with force‐free model fit parameters and so will be explored below.

### 4.1 Distribution of Properties

**Figure 5:**
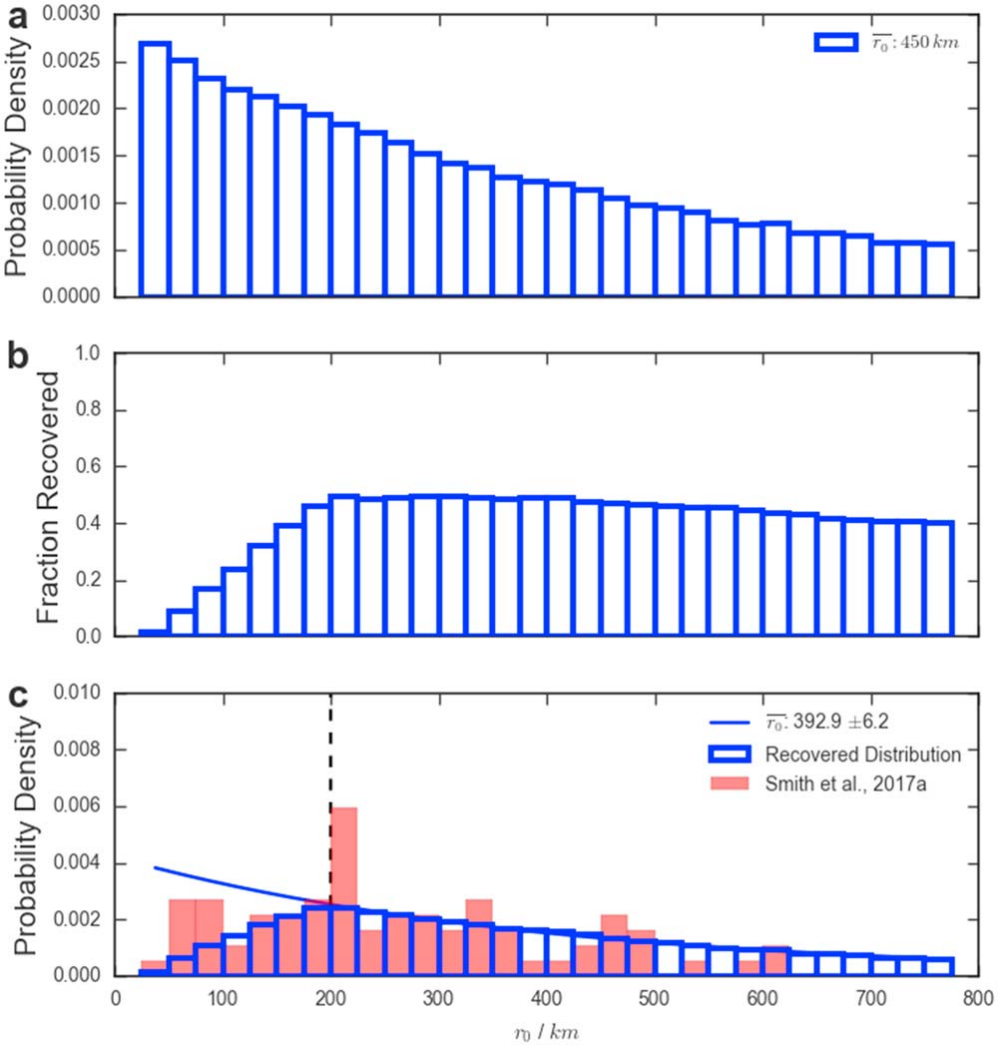
Panel (a) shows a randomly generated distribution of radii drawn from an exponential distribution with a mean of 450 km. Panel (b) shows the recovery fraction of flux ropes as a function of radius with the criteria of Smith, Slavin, Jackman, Poh, et al. (2017), while panel (c) shows the resulting distribution of radii that would be observed. In panel (c) the observations of Smith, Slavin, Jackman, Poh, et al. (2017) are provided in red as an example. The solid blue line shows the results of exponential fits to the tail of recovered distribution (r> 200 km: represented by the vertical black dashed line).

Figure 5 demonstrates the impact of selection effects on an intrinsic distribution. Figure 5a shows a synthetic distribution of flux rope radii, where the distribution has been drawn from an exponential with a mean of 450 km. The exponential distribution was chosen as appropriate from the modeling work of Fermo et al. (2010), while the choice of mean is consistent with previous work on Hermean magnetotail flux ropes (DiBraccio et al., 2015). Figure 5b shows the fractional recovery of flux ropes as a function of radius (Figure 3f), given the selection criteria employed by Smith, Slavin, Jackman, Poh, et al. (2017). Therefore, accounting for selection effects (combining Figures 5a and 5b) would result in the observed distribution shown in Figure 5c (in blue). This distribution is consistent with that observed by previous studies: displaying an exponential tail at larger radii and a drop off at the smallest spatial scales (e.g., Fear et al., 2007; Fermo et al., 2011). The results obtained by Smith, Slavin, Jackman, Poh, et al. (2017) are shown in red in Figure 5c for context, though it should be noted that their estimates were obtained using an average Hermean plasma sheet Alfvén velocity (and not the actual flux rope velocity).

Previously, studies have fitted the tail of the distribution of observed flux rope radii to an exponential function ( $\propto e^{-r/r_{0}}$; e.g., Akhavan‐Tafti et al., 2018; Fermo et al., 2011). Following this procedure, if the tail of the observed distribution in Figure 5c (i.e., *r*> 200 km) is fitted to an exponential function, the mean radius that may be inferred from the fit is $\bar{r_{0}}$ = 392.9 ± 6.2 km. This result is not consistent with the original mean of the generated distribution ( $\bar{r_{0}}$ = 450 km). Therefore, for this intrinsic distribution (and set of selection criteria) fitting to the tail of the distribution does not appear to overcome the selection effects of the survey. However, this may not be the case for all studies and will depend strongly on the shape of the recovery distribution (i.e., Figure 5b) relative to the intrinsic distribution.

Ideally, it would be a simple process to divide the distribution of an observed property (e.g., the flux rope radius or core field strength) by the recovery fraction and thus obtain an estimate of the intrinsic distribution (i.e., to go from Figure 5c to 5a). For example, if only 20% of flux ropes with a given radius will be identified with a set of selection criteria, then the *n* flux ropes observed are representative of an intrinsic $\frac{n}{0.2}=5n$ flux ropes. If the quality of the data was sufficient then this could be done trivially.

However, the flux ropes observed by MESSENGER were identified solely upon their magnetic signature, and lack simultaneous, high cadence plasma observations (e.g., DiBraccio et al., 2015; Smith, Slavin, Jackman, Poh, et al., 2017). In this case it is perhaps not appropriate to perform the correction on the inferred radii (as they are calculated with the aid of an average Alfvén velocity). Therefore, the comparisons should be made between the modeled and observed durations.

**Figure 6:**
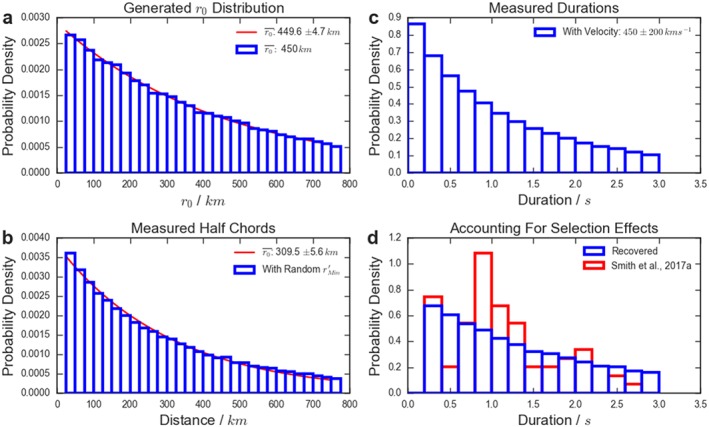
Panel (a) shows an exponentially distributed set of flux rope radii with a mean of 450 km, while panel (b) shows the distribution of radii once corrected for the impact parameter of the encounter. Panel (c) shows the duration of the signatures that would be recorded assuming a normally distributed velocity, and panel (d) shows the distribution of durations that would be observed with the selection criteria of Smith, Slavin, Jackman, Poh, et al. (2017). The solid red lines show exponential fits to the distributions in (a) and (b).

Figure 6a shows an example distribution of flux rope radii, drawn from an exponential distribution with a mean *r*_0_ of 450 km (as above). When a spacecraft passes through a flux rope it will generally not pass directly through the center of the structure, and will instead create a chord through the flux rope (assuming the structure can be approximated as a cylindrical structure and is encountered normal to its axis). Akhavan‐Tafti et al. (2018) recently highlighted the importance of correcting for this effect in statistical studies of subsolar magnetopause FTEs. Figure 6b shows the distribution of measured half chords when Figure 6a is corrected with randomly selected impact parameters ( $r_{\mathrm{Min}}^{\prime }$). Fitting this distribution to an exponential would result in the inference of a smaller mean radius than is correct (Akhavan‐Tafti et al., 2018).

Next, the distribution of half chords is converted to the duration of the magnetic signature, often defined as the time between the peaks of the bipolar signature (e.g., Kawano et al., 1992; Slavin et al., 1993). Physically this corresponds to the time between the leading and trailing edges of the flux rope. To make this conversion, the velocity of each flux rope is required. In this case, the velocities are drawn from a normal distribution with a mean of 450 km/s and a standard deviation of 200 km/s. This distribution was chosen as it is consistent with previous observations of terrestrial flux ropes (Moldwin & Hughes, 1992; Slavin et al., 2003) and measurements of the Hermean magnetotail Alfvén velocity (DiBraccio et al., 2015). The resulting distribution of durations is shown in Figure 6c. Several velocity distributions were tested and the changes were found to have a relatively small effect on the resulting distribution of durations.

Finally, the distribution in Figure 6c is sampled with the selection criteria employed by Smith, Slavin, Jackman, Poh, et al. (2017); this results in the distribution shown in blue in Figure 6d. The actual distribution observed by Smith, Slavin, Jackman, Poh, et al. (2017) is also shown in red. It is then possible to compare the results with that of Smith, Slavin, Jackman, Poh, et al. (2017) while varying the mean of the input distribution. Performing this fit allows the mean of the distribution of quasi‐force‐free flux rope radii to be calculated as $589_{-269}^{+273}$ km. While this is identified as the optimum fit, it is clear from Figure 6d that the differences between the survey and the modeled results are significant (e.g., around a duration of 1 s); this is reflected in the large uncertainties. It is likely that a larger database of events would help to clarify if this is a result of a relatively small sample size, or indicative of other properties of the system.

### 4.2 Inferred Rate

Each flux rope will occupy a unique location in the four‐dimensional parameter space described by Figures 2 and 3. In principle it would be possible to use the probability of recovering each individual flux rope as a weighting factor. Correcting each observation for the probability of its identification can then provide a rate of flux rope generation that is more reflective of the system.

**Figure 7:**
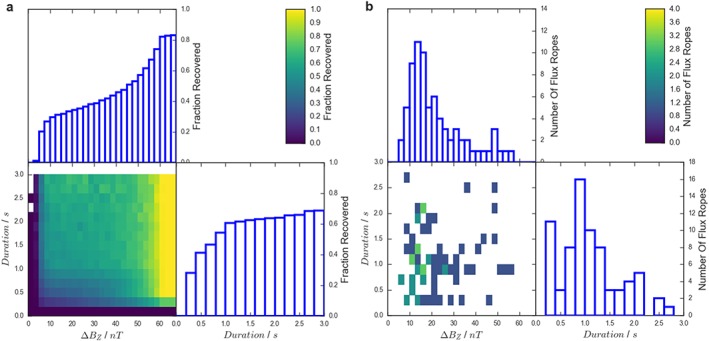
(a) The fractional recovery of model flux ropes as a function of deflection size (ΔB_Z_) and duration, for the criteria of Smith, Slavin, Jackman, Poh, et al. (2017). (b) The distribution of quasi‐force‐free flux ropes observed by Smith, Slavin, Jackman, Poh, et al. (2017).

However, for application to the survey of Smith, Slavin, Jackman, Poh, et al. (2017) it is more appropriate to apply this correction to the recovery fractions in a two dimensional parameter space described by Δ*B*_*Z*_ and the duration of the signature: Figure 7a. The reason for this is the velocity of each individual flux rope is not known, meaning the radius is not known definitively.

In total, Smith, Slavin, Jackman, Poh, et al. (2017) identified 74 quasi‐force‐free flux ropes, the distribution of which is shown in Figure 7b as a function of deflection size (Δ*B*_*Z*_) and signature duration. It is possible to divide the number of flux ropes identified (Figure 7b) by the probability that they would be identified (Figure 7a) to correct for selection effects. For example, if two flux ropes were observed with a given set of properties, but the probability that they would be identified was only 0.5 then they are likely representative of a total of four flux ropes with those combination of properties. Performing this for the full sample indicates that the 74 identifications made by Smith, Slavin, Jackman, Poh, et al. (2017) are representative of a total population of 181 quasi‐force‐free flux ropes.

The survey by Smith, Slavin, Jackman, Poh, et al. (2017) considered a total of 1,482 min of MESSENGER plasma sheet observations. With 74 observed flux ropes this corresponds to an average rate of observation of 0.05 min^−1^. Correcting each flux rope for the probability of its identification increases the total number of flux ropes, and therefore the inferred rate of flux ropes encountered in the Hermean magnetotail increases to 0.12 min^−1^. For context, the rate of terrestrial magnetotail flux rope observations is around 0.7 × 10^−3^ min^−1^ (Imber et al., 2011). Future work should involve running such terrestrial observations through the techniques described, so the inferred values can be fully compared.

## 5 Discussion

Discrepancies between modeled and observed distributions of flux ropes have suggested that current surveys of spacecraft data have not identified the complete statistical distribution of flux ropes, with small radius structures in particular being underrepresented (Fermo et al., 2011). One potential reason for this is the selection criteria placed upon potential magnetic field signatures. To address this issue a simple Monte Carlo technique has been presented that allows the evaluation and estimation of the resulting selection biases. Correcting the observed distributions allows a better estimation of the underlying properties and rate of flux rope generation.

First, we have shown that the poor recovery of flux ropes with small radii cannot be overcome by fitting an exponential model to the tail of the distribution (c.f. Fermo et al., 2011). The error involved in this process will depend on the selection criteria utilized and the underlying properties of the distribution.

The first large survey of Hermean magnetotail flux ropes with MESSENGER data inferred that their average radius was ∼345 km (DiBraccio et al., 2015). Later, Smith, Slavin, Jackman, Poh, et al. (2017) performed a survey of a larger quantity of data and inferred a mean radius of ∼262 km. Both studies used the cylindrically symmetric force‐free model to correct for the relative trajectory of the spacecraft and assumed a flux rope velocity of 465 km/s. In this work we reprocess the results of Smith, Slavin, Jackman, Poh, et al. (2017), assuming a normally distributed flux rope velocity of 450 ± 200 km/s. A Monte Carlo approach was used to find that the best fit mean flux rope radius was $589_{-269}^{+273}$ km, larger than previously inferred though associated with large uncertainties. The large uncertainties present are likely a result of the incomplete sampling, a problem that could be addressed by future, larger studies. The size distribution of flux ropes has implications for their generation and coalescence (Fermo et al., 2011).

Finally, accounting for flux ropes present (but not identified) increases the inferred rate of flux rope generation in the Hermean magnetotail by a factor greater than two to ∼0.12 min^−1^. This has implications for the formation of flux ropes, as well as the total mass and magnetic flux contained within such structures. In context, however, the flux contained within the average flux rope (0.003 MWb; Smith, Slavin, Jackman, Poh, et al., 2017) is small compared to the average change in open flux during a Hermean substorm (0.69 ± 0.38 MWb; Imber & Slavin, 2017).

## 6 Conclusions

Surveys of spacecraft magnetometer data can be useful to assess the properties, location, and recurrence of reconnection related structures within planetary magnetotails. These in turn can provide information regarding the conditions at the reconnection site and the dynamic nature of the magnetotail. However, even large spacecraft surveys are restricted by the orbital sampling of the spacecraft and the criteria placed upon the signatures of the event in question. Ultimately, the selection criteria employed can filter through the analysis and affect the conclusions of the study. We have presented a Monte Carlo method of estimating the fraction of events that are observed, as a function of various underlying parameters of the flux ropes. The effects of orbital sampling are considered in a companion study (Smith et al., 2018).

The evaluation of the fractional recovery of flux ropes can allow the observed distributions of properties to be corrected, providing insight into the underlying distributions present. An application of this has been shown with regards to the distribution of flux rope radii observed in the Hermean magnetotail. In this case, the subsequent fit is made to the distribution of durations observed (due to the lack of simultaneous high resolution plasma data). The most consistent result is found with a distribution with a mean radius of $589_{-269}^{+273}$ km.

Finally, each individual identification can be corrected for the likelihood that it would be made. For example, small flux ropes may be underrepresented as they are more difficult and thus less likely to be identified. Following this, the 74 quasi‐force‐free flux ropes observed by Smith, Slavin, Jackman, Poh, et al. (2017) are indicative of a total population of 181 flux ropes. This has the effect of increasing the overall rate of flux ropes in the Hermean tail from 0.05 to 0.12 min^−1^, a value that is approximately 180 times that previously observed in the terrestrial magnetotail, indicating the hugely dynamic nature of the Hermean magnetotail.
